# Opportunities and challenges for plague vector control in Madagascar

**DOI:** 10.1371/journal.pntd.0013054

**Published:** 2025-05-13

**Authors:** Annick Onimalala Raveloson, Mireille Harimalala, Beza Ramasindrazana, Romain Girod, Thomas Robert Gillespie, Diego Ayala, Adelaide Miarinjara

**Affiliations:** 1 University of Antananarivo, Antananarivo, Madagascar; 2 Institut Pasteur de Madagascar, Antananarivo, Madagascar; 3 Departments of Environmental Sciences and Environmental Health, Emory University and Rollins School of Public Health, Atlanta, United States of America; 4 Centre ValBio, Ranomafana, Fianarantsoa, Madagascar; 5 UMR MIVEGEC, University of Montpellier, CNRS, IRD, Montpellier, France; University of Texas Medical Branch, UNITED STATES OF AMERICA

## Abstract

Plague is a rodent-borne disease transmitted to humans by the bite of fleas infected with the bacterium *Yersinia pestis*. Flea control is a key part of the overall plague management strategy. Insecticide-based strategies are designed to reduce or eliminate fleas from the environment in order to stop the disease transmission cycle during outbreaks. Many efforts have been directed toward monitoring flea susceptibility to insecticides using standardized bioassay methods recommended by the World Health Organization (WHO). Several studies have reported the development of insecticide resistance in vector species across Madagascar, which could be one of the factors contributing to the re-emergence of plague in endemic foci. However, the assessment of the operational efficacy of vector control in the field has received less attention. Furthermore, the mechanisms conferring flea resistance to insecticides remain poorly explored. In this review, we summarize the current understanding of (i) the effectiveness of insecticides for flea vector control in Madagascar, (ii) longitudinal surveillance of insecticide resistance in flea vector populations across the country, and (iii) insecticide resistance mechanisms in these fleas. Current vector control methods, including WHO standard methods for assessing the susceptibility or resistance of adult fleas, are presented and discussed. In addition, we propose recommendations for future research to improve the effectiveness of vector control and insecticide resistance mitigation for more effective control of plague-vector fleas in Madagascar.

## Background

### Epidemiology overview

Plague is a zoonotic disease caused by the bacterium *Yersinia pestis*, primarily affecting rodents and human infection remains accidental [1]. The most common route of transmission occurs through the bite of infected fleas that act as vectors between plague-infected rodents and humans. Humans are susceptible to plague and typically develop bubonic plague following infected flea bites. Secondary pneumonic plague can lead to airborne transmission of the infective agent via the respiratory route, potentially resulting in primary pneumonic plague among close human contacts. Despite available antibiotic treatments, plague continues to pose a public health threat in some countries including Madagascar.

Madagascar has more than one century history of battle against plague disease since the first record of human cases in 1898 during the third global plague pandemic [2]. The harbor town of Toamasina was then first affected with 288 cases, including 97 deaths and by 1921, the epidemic reached the capital, Antananarivo, in the Central Highlands [3]. By 1932, cases increased from 1,000 to nearly 4,000 per year. After sharp declines in 1936 and 1953–1954, the number of cases remained under 100 annually from 1954 to 1988 due to effective mitigation measures [2]. However, between 1989 and 1995, 500–1,300 suspected cases were reported annually, partially due to the availability of improved diagnostic tools [4,5]. From 2004 to 2009, Madagascar accounted for 30% of human cases worldwide with 1,214 cases and 98 deaths reported. Between 2010 and 2015, 200–700 suspected cases of plague were reported annually and approximately 55% of these cases were laboratory confirmed [6]. Madagascar reported 80.5% of all human cases in the world between 2015 and 2018 [7], with 395 bubonic plague cases reported in 2017 [6]. Bubonic is the dominant clinical form of plague in Madagascar, highlighting the significant role of fleas in the pathogen transmission and disease spread. Bubonic plague consistently accounts for a high proportion of reported cases: 75–92% before 1990, 97% between 1980 and 2001 [8], and 86.6% of suspected cases from 2007 to 2011 [9]. Between 1998 and 2016, it represented 93% of both confirmed and presumptive cases [10]. The pneumonic form remains less dominant but can lead to a severe health crisis when it spreads in urban settings, as seen in 2017 [6]. Plague is endemic in rural areas and cases have been predominantly reported in regions with elevation above 800 m, making the Central Highlands the primary focus areas, along with some northern regions with mid to high elevations [5,9]. Plague disappeared from the coastal areas, however, a focus persists in the harbor town of Mahajanga on the west coast of Madagascar [11]. Human plague cases are mostly recorded from October to April in the Central Highlands, corresponding to the hot and rainy season, whereas from July to November in Mahajanga[9].

### Ecology of plague vectors and reservoirs

Worldwide, more than 80 flea species are known to be involved in human plague transmission [12], and far more flea species are parasitizing mammals involved or suspected to be involved in the zoonotic cycle [13]. Recent studies have identified 363 mammal species and five bird species as susceptible to plague. [13].

In Madagascar, the main domestic reservoir and hosts for plague are the black rat (*Rattus rattus*), the brown rat (*Rattus norvegicus*), and the Asian house shrew (*Suncus murinus*) [9]. The black rat is known to be the main reservoir in the rural areas. Some small wild mammal reservoirs are suspected to play a role in plague maintenance in the Central Highlands [14,15]. The fleas *Xenopsylla cheopis* and *Synopsyllus fonquerniei* are considered the primary vectors in Madagascar [5,9,16].

The Oriental rat flea *X. cheopis,* commonly parasitizes commensal rats (*R. rattus* and *R. norvegicus)* caught indoors and is notably abundant during the hot season which coincides with the plague transmission season in the Central Highlands [3]. The endemic flea *S. fonquerniei* is more generally found on the fur or within burrows of rodents living outside houses but can also infest wild small mammals (Tenrecidae, Nesomyinae) [9,17–19]. Ecological studies suggested that *S. fonquerniei* is most abundant at elevation above 800 m and reached its abundance peak during September–November [17,18,20].

More than 40 flea species have been described in Madagascar, and the involvement of other species in plague transmission requires investigation [21]. Most notably, the human flea, *Pulex irritans*, has been found naturally infected by *Y. pestis* during plague outbreaks [22], and *X. brasiliensis*, a key vector in East Africa, has been found in Madagascar, although not observed naturally infected in the island yet [23]. Further research is needed to establish the vectorial capacity of fleas such as *S. fonquerniei*, *P. irritans* and *X. brasiliensis*, as well as the role played by sylvatic fleas in the maintenance of zoonotic plague.

### Overview of plague vector control in Madagascar

Targeting the vectors that transmit disease is an effective preventive approach against most vector-borne diseases. Interventions that reduce human-vector contact and vector density and/or survival can suppress and even halt transmission [24]. Chemical intervention has been recurrently used for plague control and prevention in Madagascar. The goals are to reduce rapidly the density of flea vectors and to interrupt the transmission chain. The flea index (FI), the mean number of flea vectors collected per host (i.e., small mammals), is the primary index used to evaluate infected-flea exposure risk [1,25]. A specific flea index (SFI) can be used when focusing on particular flea species. For instance, a *X. cheopis* SFI > 1 is a risk indicator during the plague transmission season in areas where plague is endemic [1]. Insecticide dusting methods, where insecticide powder is applied to burrows entrance and places frequented by rodents, is recommended by WHO to control rat fleas during plague outbreaks [26,27].

**Fig 1:**
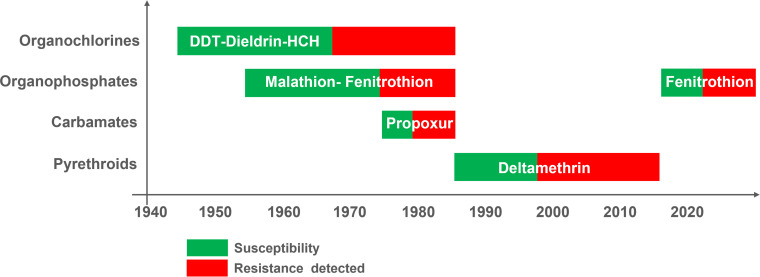
History of *Xenopsylla cheopis* insecticide resistance in Madagascar (Modified and updated from Chanteau, 2006) [33].

Residual contact insecticides for plague control were introduced for the first time in Madagascar in 1947 [28]. Their widespread and systematic use was responsible of an unprecedented reduction of flea density and diminution of reported human plague cases in the capital Antananarivo [3,29,30]. Dichlorodiphenyltrichloroethane (DDT) was the first insecticide used against plague vectors in 1947 (Fig 1). Later, in addition to DDT, hexachlorocyclohexane (HCH), dieldrin, malathion, deltamethrin and fenitrothion were used in response to plague epidemics [16,29,31,32]. The history of insecticide uses and first detection of *X. cheopis* insecticide resistance in Madagascar is presented in Fig 1.

A detailed account of vector control measures developed by health authorities to curb human cases in Antananarivo around 1949 reported that insecticide powder (a mixture of DDT and HCH) was systematically spread as a prophylactic measure every six months and included all buildings of the district [28]. In addition, during plague transmission season, neighborhoods deemed at risk received additional treatments. Finally, insecticide treatment was mandated in households with suspicious deaths. In 1950, a total of ten tons of HCH and ten tons of DDT were used, with an average consumption of 400 kg of insecticide per week [28]. This campaign reduced the average *X. cheopis* index from 8.0 to 9.0 to 0.3 and resulted in SFI below 1.0 until 1960 [3]. Despite such successes in the capital city, this intensive and costly protocol could not be implemented on a larger scale. Instead, vector control was only deployed nationally in response to epizootic infection or suspicion or confirmation of human cases. Medical inspectors responsible for districts in endemic areas were instructed to treat all habitations within a 25 km radius of any threat of plague, whether human cases were reported, or dead rats were observed [3]. In the 1970s, villages in plague-endemic areas were required to have 25 kg of insecticide as a plague epidemic preparedness measure [29]. While these instructions were given as an indication, a treatment radius of the patient’s dwelling and of other dwellings within 200 m were later recommended by WHO [27].

Monitoring for insecticide resistance in rat fleas started in 1950s [3], and demonstration of resistance to DDT in 1960 compelled health authorities to regularly shift to new active ingredients for plague control (Fig 1) [29]. Deltamethrin was designated by the National Plague Control Program (NPCP) to control flea populations as the primary response intervention in 1990s [32]. Since 1998, several populations of fleas were found to be resistant to deltamethrin [34,35]. A more extensive survey showed that most tested populations of *X. cheopis* were resistant to deltamethrin [32]. Following a study showing that fenitrothion gave the highest mortality rate in all populations tested, this active ingredient was proposed as an alternative to deltamethrin [36]. Currently, fenitrothion powder is used indoors in response to plague outbreaks in Madagascar [16,37].

### Review aims and objectives

Major improvements have been made in terms of plague diagnostics, treatments, and human case management during the last decades [38]. However, and despite the prominent role in the disease transmission, the same approach and methods have been used for vector control since the colonial era (from the 1940s) in Madagascar. Given the major role of fleas in plague transmission, and with the bubonic form predominant in Madagascar, improving flea vector control knowledge should be a priority. The goal of this review is to examine the history of flea vector control in Madagascar until 2023, focusing on the development of insecticide resistance in flea vectors and its mechanisms. We highlight and discuss the effectiveness of the tools and strategies employed and provide recommendations for future research direction to improve vector control and insecticide resistance monitoring in *Y. pestis* vectors.

## Database extraction methods

### Study design and search strategy

**Fig 2:**
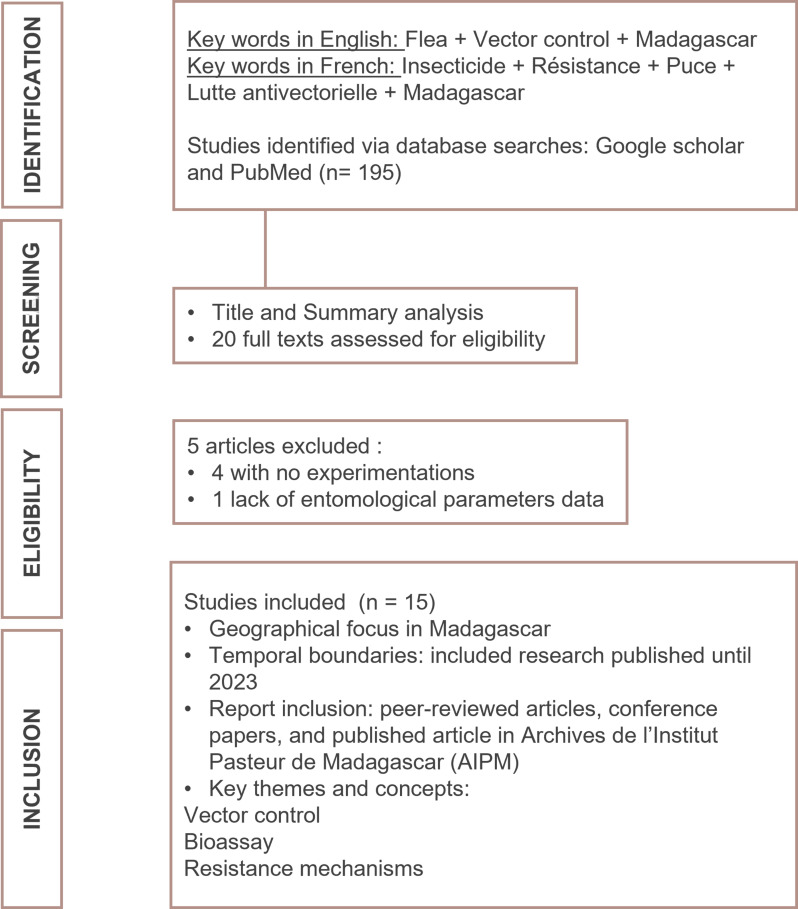
Selection process for eligible publications.

A systematic search of published literature using online scientific bibliographic databases was performed using the following key words in English and French: insecticide resistance, flea, vector control, Madagascar, resistance mechanisms. Databases included PubMed, Google Scholar, and the Archives de l’Institut Pasteur de Madagascar (Fig 2).

### Study eligibility

For the review, we included relevant published data following these criteria:

- Geographical focus: focused on studies conducted in Madagascar, considering the unique history of plague mitigation that may influence insecticide resistance in plague vectors in this country.
- Temporal boundaries: included research published until 2023 to provide a contemporary overview of the evolution of knowledge on vector control and insecticide resistance in Madagascar.
- Key themes and concepts: deployed or experimental vector control strategies and their efficacy evaluation, surveillance of phenotypic resistance, mechanisms of insecticide resistance experimentation, resistance prevalence rates and geographic variations of insecticide resistance, and implications of resistance on current and future plague control efforts in Madagascar.
- Report inclusion: peer-reviewed articles, conference papers, and reports that specifically address vector control evaluation and insecticide resistance surveillance in plague vectors in Madagascar available as full text online. We excluded reports describing vector control during plague outbreak, publications reporting only previously published results, and studies not directly related to the topic or conducted outside the specified geographic and temporal boundaries.

### Data extraction and quality assessment

Two reviewers separately extracted data, and disagreements were resolved via discussion. Data was entered into Microsoft Excel datasheets. For laboratory or field insecticide assays, if flea numbers and mortality rates were not reported in the publication, efforts were made to contact authors to obtain this information. Geo-referencing followed decimal degrees format using the set of coordinates or location names provided in publications. The maps were generated with QGIS software (https://qgis.org/en/site/about/index.html). Administrative boundaries were downloaded from GADM (https://gadm.org/index.html).

#### Vector control assessment database.

Vector control studies evaluated the impact of insecticide applications on flea populations. The effectiveness of interventions was assessed using flea index (FI) values. Flea index values were compared between prior and post intervention values or between values obtained from control plots. In this review, we assessed the success of vector control interventions using two criteria:

(1) The ability of the intervention to induce a statistically significant decrease in FI compared to baseline value and/or control plots.
(2) The ability of the intervention to reduce the specific FI below one, the desired outcome for an intervention in response to a plague epidemic.

For each vector control assessment, the following were included in the dataset: first author, institution, publication year, GPS (Global positioning system) coordinates, location, vector control method, flea index before treatment, flea index for each time point after treatment, vector species, commercial insecticide (if applicable), active ingredient, concentration, family, formulation, and reported treatment effectiveness.

#### Flea susceptibility assay database.

Susceptibility assays measured flea population response (mortality) to known active ingredient concentration(s) during a diagnostic time, to establish susceptibility or resistance status through controlled laboratory experiments. Studies that reported only validation of diagnostic time and diagnostic concentrations were not included. For each insecticide susceptibility assay, the following were included in the analysis: first author, publication year, GPS coordinates, institution, number of fleas tested, flea species, bioassay method, insecticide, concentration and family, mortality rates and susceptibility status. Flea status regarding insecticide was assigned based on WHO criteria: resistant (mortality <80%), tolerant (mortality from 80 to 97%), and susceptible (mortality from 98 to 100%) [39].

## The plague vector control toolbox

### Flea control using insecticide dust formulations

DDT was the first chemical insecticide used for flea control, and the promising results obtained with DDT dust or emulsion made it an insecticide of choice to mitigate flea-borne diseases such as plague [40]. The plague monograph published by the WHO in 1954 discussed the pros and cons of using liquid or powder forms for controlling rodent fleas [25]. DDT powder was preferred and considered more practical because sprayed materials adhered to household surfaces, whereas dust deposits adhered to rodent fur and consequently were transported to burrows and nests. However, the efficacy of insecticide dust can be limited at time due to unavailability of application tools, toxicity and disapproval by target population [41]. A primary concern is the risk of inhalation of pesticide particles smaller than 10 µm in diameter [27]. WHO guidelines list several active ingredients formulated as powder to be used against adult fleas and larvae [26,27]. Several insecticides were used in Madagascar for flea control, and transitions to new active ingredients usually followed detection of resistance (Fig 1). DDT, deltamethrin and fenitrothion were certainly the most extensively used.

### Insecticide dust used in bait stations

To control outdoor rodent fleas, strategies involved bait stations to use hosts to transport insecticide into burrows and other hard-to-reach places [42,43]. In Madagascar, this approach has been updated to include rodenticide, in addition to insecticide powder, to achieve simultaneous reservoir and vector control [44–46]. The use of a slow acting rodenticide ensures control of fleas before the host rodent succumbs to the toxic bait. The bait stations have the merit of not requiring spraying equipment or specific experience. By capitalizing on a good knowledge of rodent movement [47], this strategy reduces the quantity of insecticide used and the risks of poisoning and contamination as the insecticide is confined in the station. Since the efficacy of this method to rapidly eliminate fleas remains unknown [37], its use as vector control against plague needs further study.

### Flea control using liquid formulations

Insecticide dusting remains the primary method recommended by WHO for managing rat fleas during plague epidemics, but the use of liquid formulations has been documented in Madagascar [31]. While rare, new products with different formulations have been tested to mitigate the development of insecticide resistance and to improve the efficacy of treatments [48]. Liquid formulations have been widely used against malaria vectors, whose distributions sometimes overlap with plague-endemic areas. In Madagascar, successful simultaneous vector control for both diseases using different insecticide formulations has been reported [28]. However, the specific impact of each formulation on insect targets has not been thoroughly evaluated.

The efficacy of malaria indoor residual spraying on rodent fleas has been reported elsewhere [49], supporting an integrated vector control approach for both endemic diseases. In the West Nile Region of Uganda, indoor residual spraying is used by authorities for flea control in response to human or rodent plague cases, although its deployment as preventive measure raised many challenges [50]. The topical application of liquid insecticide showed promising results in the same region, capitalizing on the knowledge of indoor rodents’ movement while minimizing environment contamination by the use of insecticide delivery tubes [51]. In some instances, community engagement can play a key role in the selection of insecticide for plague vector control. Despite not being specifically formulated for flea control, the product called ‘Ant Killer’ was preferred by community members due to its perceived effectiveness and was used to spray more than 28,000 households in response to a bubonic plague outbreak in Democratic Republic of Congo [52].

### Systemic insecticides

Systemic insecticides are particularly effective against fleas when ingested in host blood and pose minimal risk to the environment [53]. Deployment requires no special equipment like insecticide spreaders or bait stations, making it more manageable in emergency situations. Despite extensive use of systemic insecticides against fleas infesting pets [54] and wild rodents [55,56], this method has rarely been employed for controlling fleas on commensal rodents. A field trial conducted in Uganda with imidacloprid, although innovative, raised several operational challenges [57]. In Madagascar, a pilot study with the systemic insecticide fipronil yielded promising results [58]. This study aimed to expand the range of vector control tools for plague management in Madagascar and to explore new active ingredients in response to increasing prevalence of insecticide resistance. Commercially available formulations containing systemic insecticides have proven effective in other regions [59,60]; however, a carefully planned field trial is necessary to evaluate their feasibility as a vector control method in the specific context of Madagascar’s plague ecosystem with consideration of the behavior of rodents and humans.

## Plague vector control research in Madagascar

Research plays an important role in the selection of appropriate vector control strategies. While recommendations from organizations such as WHO or WHOPES (World Health Organization Pesticide Evaluation Scheme) provide guidelines, prioritizing research for evidence-based interventions empowers countries to tailor their vector control methods and tools to local conditions, assess their effectiveness, adapt them to changing circumstances and optimize resource allocation. Research regarding plague vector surveillance, prevention and control in Madagascar can be divided into three categories: (1) evaluation of current and new methods and tools through field trials, (2) monitoring of insecticide susceptibility, and (3) study of resistance mechanisms.

### Evaluating plague vector control methods in real-world settings

The population dynamics of plague vectors and reservoirs are subject to changes over time due to various factors such as climate change, urbanization, shifts in public health policy, and the development of insecticide resistance. These factors can significantly influence the patterns of disease transmission. In particular, the emergence of insecticide resistance poses a constant concern, potentially compromising the effectiveness of insecticide-based vector control interventions. To address this challenge, operational efficacy of these interventions, determined through field trials, is a focal point of research efforts discussed in this review. This research aims to assess the effectiveness of different insecticide formulations, application methods, and integrated vector and reservoir management approaches. By evaluating the performance of various control strategies in real-world settings, researchers seek to identify the most efficient and sustainable approaches for managing plague vectors and reducing disease transmission. Much of this research has shaped the current state of plague vector control in Madagascar. In total, we found five publications reporting results of vector control field evaluations (Table 1). All data on flea vector control were generated by the Institut Pasteur de Madagascar (IPM). These studies explored the effects of vector control interventions using different approaches, categorized as (1) indoor residual spraying, (2) powder dusting, and (3) use of bait stations.

**Table 1:** Summary of publications reporting results from field evaluations of insecticides in Madagascar from 1960 to 2017.

Ref.	Insecticide	Concentration	Formulation	Flea index^a^										
				Day 0	Day 2	Day 6	Day 7	Day 30	Day 60	Day 90	Day 120	Day 150	Day 180	Day 210
[31]	Dieldrin + malathion	^b^	Liquid spray	3.4	_	_	_	0.1		0.0	_	_	_	0.7
	Dieldrin + malathion	^b^	Liquid spray	3.2	_		_	0.5		0.8	_	_	_	1.1
[61]	Deltamethrin	2 g/kg	Powder	6.1	_	_	3.8	_	_	_	_	_	_	_
	Pyrimiphos-methyl^c^	20 g/kg	Powder	17.3	_	_	3.2	3.2	_	_	_	_	_	_
	Bendiocarb^c^	10 g/kg	Powder	17.3	_	_	3.2	3.2	_	_	_	_	_	_
[48]	Deltamethrin	2 g/kg	Powder	1.9	_	_	_	0.4	1.7	_	_	_	_	_
	Diazinon^c^	240 g/l	Liquid spray	2.5	_	_	_	1.1	2.4	_	_	_	_	_
[46]	Propoxur	30 g/kg	Bait station^d^	_	_	_	_	1.2	7	0	0	0	0	_
[37]	Fenitrothion	20 g/kg	Bait station^e^	1.0	0.7	_	_	2.3	_	_	_	_	_	_
	Fenitrothion	20 g/kg	Bait station^e^	1.5	1.0	_	_	_	_	_	_	_	_	_
	Fenitrothion	20 g/kg	Bait station^d^	1.7	1.5	0.8	_		_	_	_	_	_	_
	Fenitrothion	20 g/kg	Powder	0.5	0.2	_	_	0.7	_	_	_	_	_	_
	Fenitrothion	20 g/kg	Powder	1.7	0.3	0.3	_	_	_	_	_	_	_	_

^a^Specific flea index for *X. cheopis* in [48,61] and [46]. For [31] and [37] the flea index concerns all species confounded.

^b^Emulsion containing 18.5% of dieldrin and 15% of malathion.

^c^Insecticides that were not used by the National Plague Control Program.

^d^More than one bait station per household.

^e^Single bait station per household.

Ref.: Reference.

#### Direct measurement of vector control effectiveness.

Establishing real-world efficacy of plague vector control is challenging, particularly due to difficulty in obtaining flea indices or infestation rates and susceptibility status before outbreaks. Evaluation of insecticide treatment requires field trials and pilot studies. The sharp decrease in the rat flea index and human plague cases in Antananarivo following intensive DDT treatment in the 1950s was cited for decades to illustrate the success of vector control [29]. The resulting low incidence of human plague however may have been the consequence of other measures including sanitation, antibiotic treatments, and vaccination.

An exceptional instance of directly measuring vector control effectiveness emerges from a year-round ecological survey conducted in a typical village situated within a plague-endemic area of Madagascar [31]. The specific context of plague control policy in the 1950s, involving preventive insecticide treatment in endemic regions, allowed investigators to record entomological parameters at multiple time points both before and after insecticide treatments. From April 1958 to June 1959, every two months, investigators trapped rodents and collected fleas in several locations within the village, while routine plague vector control treatments occurred in November 1958. The flea index recorded in October 1958 can be considered a baseline, and the flea index recorded in December 1958, the post intervention value. The flea index dropped from 3.4 to 0.1 for indoor rodents and from 3.2 to 0.5 for outdoor rodents. These values remained below one until the end of the observation period, more than six months after treatment for indoor rodents, which demonstrated the effectiveness of vector control (Table 1).

#### Evaluation of the effectiveness of ongoing vector control strategy using field trials.

The efficacy of insecticide treatment, simulating a response to a plague outbreak, was measured in three independent field trials and involved the insecticides that were then used routinely for vector control, namely deltamethrin and fenitrothion [37,48,61]. The ability of each insecticide treatment in reducing the FI was usually assessed before and after treatment (Table 1). The objective of these experiments was to test the efficacy of the standard method against new products or application method. This approach aimed to explore alternative solutions, thereby expanding the range of options available.

In the first study, the investigators demonstrated that deltamethrin could not achieve a significant drop in flea index after seven days [61]. Besides, the expected outcome from treatment deployed for plague outbreak mitigation is the reduction of the FI to a value below one. According to the authors hypothesis, unsatisfactory results obtained with deltamethrin could be linked to flea population resistance, which was previously demonstrated through bioassays [34].

In the second study, the investigators wanted to test the effectiveness of a new active ingredient (diazinon) under a new formulation (microencapsulation, 240 g/L) against the adopted deltamethrin powder (0.2%) [48]. They reported a similar efficacy for the two products, with a significant decrease in FI after 30 days. Unfortunately, the immediate effect required in the context of plague emergence was not evaluated despite the importance of this information for public health.

The third study reported satisfactory results obtained with fenitrothion powder, reducing significantly the FI two days after treatment to a value below one [37]. This study demonstrated that effective vector control was achieved in a short period of time, but information on remanence was lacking. While most all the results supported the continuation of the current vector control policy in Madagascar, additional research has been conducted to examine the feasibility of methods that could sustainably prevent human cases, by simultaneously reducing the density of both vectors and reservoirs.

#### Field trials with bait stations for flea and rodent control: findings and future directions.

The first pilot study using bait stations known as “boîte de Kartman” yielded satisfactory bait consumption rates and reduction of the rodent population in treated areas after one month of sustained use [46]. However, the study design did not allow for accurate monitoring of immediate flea elimination, which is crucial during a plague epidemic (Table 1). Establishing a flea index before the toxic effects of the rodenticide would have been a more effective way to confirm the timely death of fleas prior to the elimination of their rodent hosts.

In 2019, a different study design was implemented to evaluate the ability of similar bait stations (“boîte de Kartman”) to reduce the flea index over a short period of time [37]. After two nights of deployment, bait stations with only insecticide powder did not achieve a significant decrease in flea index compared to pre-treatment values and untreated control village values. Increasing the number of bait stations also did not improve their efficacy (Table 1). Therefore, bait stations with insecticide powder alone did not provide effective and immediate flea elimination, raising concerns about their effectiveness as a vector control tool during an epidemic.

However, when combined with rodenticide and other reservoir control approaches, this method showed potential as a preventive measure to decrease risk indicators values and reduce plague incidence, as demonstrated in a recent study [45]. Yet, the effectiveness of bait stations in eliminating fleas before the death of rodents needs further investigation [37,44].

## Monitoring the status of insecticide resistance

### Bioassay protocol on flea

#### History of bioassay protocol on rat flea.

Phenotypic susceptibility tests were developed primarily to detect physiological insecticide resistance in the rat flea population [39,62]. Prior to 1960, there were few records of rat flea resistance to insecticide, like the operational failure of DDT powder to achieve flea control in Ecuador [63]. Furthermore, the standard method for measuring flea susceptibility to insecticide was lacking [64,65]. The provisional method proposed by WHO in 1960 was largely inspired by the method used by investigators in India [64], using insecticide-treated papers designed for malaria vectors, cut in strips that can fit inside test tubes [62]. Between 1960 and 1970, the configuration of the insecticide paper cuts changed from a folded strip (Z shape) to a vertical paper strip tapered at one end [39].

The WHO flea bioassay exposed fleas to a series of insecticide concentrations and recorded the lethal concentrations (LC or lethal dose, LD) giving 50% and 90% mortality [62]. The proposed exposure time was one hour, followed by 24 h holding time and LC (or LD) was determined graphically by log-probit method. If low mortality was obtained with the higher concentrations, it was recommended to perform a 24 h exposure, without observing the holding time. Once the dose-mortality curve was established in the susceptible population, resistance surveillance would be performed on a single concentration, called the diagnostic concentration, which was the lowest consistently giving 100% mortality. In later version of the protocol, this diagnostic concentration became the double of the lowest concentration giving 100% mortality [39,66].

The criteria for this bioassay for flea exposure to a single diagnostic dose followed those developed for a bioassay for *Anopheles* sp. exposure to DDT [67]. According to these criteria, a mortality rate above 98% signifies susceptibility to the tested insecticide. A mortality rate between 80 and 98% signifies that verification is required to confirm resistance (later, categorized as “tolerant”), and finally, a mortality rate below 80% means that tested flea population is resistant. As these criteria are influenced by abiotic and biotic factors, they require standard laboratory conditions and a uniform population [39].

In 1976, the WHO Expert Committee on Insecticides recommended investigation of specific diagnostic doses for fleas [68]. Provisional diagnostic doses for flea have been published in WHO reports with a relatively long exposure time (ranging from 1 h 25 min to 24 h) for several insecticides like DDT, dieldrin, propoxur, fenchlorphos, malathion, fenitrothion and trichlorphon [67,69,70].

Since the use of synthetic pyrethroids for public health interventions was still uncommon, a baseline study was undertaken in Madagascar to determine the diagnostic exposure time for deltamethrin. This initial bioassay was performed using insecticide-treated paper containing deltamethrin at 0.025% provided by WHO. Investigators at IPM used laboratory strains of *X. cheopis* and *S. fonquerniei* maintained for several years without insecticidal pressure, and established diagnostic time of eight hours for deltamethrin, which has become the standard diagnostic time for all pyrethroids (Table 2) [30]. Interestingly, a baseline study for diagnostic dose was not reported on *X. cheopis,* and 0.025% deltamethrin matched the tentative diagnostic dose for *Anopheles and Culex quinquefasciatus* mosquitoes published earlier [67]. Two hypotheses have been proposed about the long exposure time for deltamethrin, the first is attributed to *a posteriori* development of resistance with this insecticide in flea populations [34], while the second is attributed to cross-resistance after exposure to DDT treatment [32]. However, the selection of populations for the experiment could also have had a major impact on the results. The WHO standard guidelines for establishing diagnostic dose or diagnostic exposure time recommended the use of a susceptible reference population, never subjected to insecticidal pressure [71]. Using resistant population to establish diagnostic dose could have a major impact on bioassay results. The results of tests to determine the diagnostic exposure time to DDT, dieldrin, malathion and propoxur were inconclusive, with an exposure time more than 48 h for malathion, nine days for DDT and dieldrin and 10 h for propoxur [67].

**Table 2:** Summary of *Xenopsylla cheopis* laboratory assay published from 1998 to 2022.

Insecticides	Diagnostic dose (%)	Diagnostic time (h)	Total bioassay	Resistant		Tolerant		Susceptible	
				Bioassay	Proportion (%)	Bioassay	Proportion (%)	Bioassay	Proportion (%)
*Pyrethroids (PYs)*									
Total PY	_	_	146	114	79.1	26	17.1	6	3.8
Alphacypermethrin	0.025	8	14	14	100.0	0	0.0	0	0.0
Cyfluthrin	0.15	8	18	6	33.3	10	55.6	2	11.1
Etofenprox	0.5	8	14	14	100.0	0	0.0	0	0.0
Deltamethrin	0.025	8	6	3	50.0	3	50.0	0	0.0
Deltamethrin	0.05	8	53	45	84.9	6	11.3	2	3.8
Permethrin	0.25	8	2	2	100.0	0	0.0	0	0.0
Permethrin	0.75	8	22	12	54.6	8	36.4	2	9.1
Lambda cyhalothrin	0.05	8	3	3	100.0	0	0.0	0	0.0
Lambda cyhalothrin	0.1	8	14	14	100.0	0	0.0	0	0.0
*Organophosphates (OPs)*									
Total OP	_	_	43	19	31.6	12	39.1	12	29.3
Fenitrothion	1	5	25	13	52.0	7	28.0	5	20.0
Malathion	5	5	14	6	42.9	2	14.3	6	42.9
Pyrimiphos-methyl	0.9	5	2	0	0.0	2	100.0	0	0.0
Pyrimiphos-methyl	2	5	2	0	0.0	1	50.0	1	50.0
*Organochlorines (OCs)*									
Total OC	_	_	35	21	50.0	3	10.7	11	39.3
DDT	1	24	3	3	100.0	0	0.0	0	0.0
DDT	4	6	18	18	100.0	0	0.0	0	0.0
Dieldrin	4	6	14	0	0.0	3	21.4	11	78.6
*Carbamates (CMs)*									
Total CA	_	_	38	28	73.9	2	5.0	8	21.1
Bendiocarb	0.1	5	14	14	100.0	0	0.0	0	0.0
Bendiocarb	1	5	6	0	0.0	2	33.3	4	66.7
Propoxur	0.10	5	18	14	77.8	0.00	0.0	4.00	22.2

#### Description of the current bioassay protocol on flea.

Considering the fact that fleas are ectoparasites, the first step was trapping rodent hosts to collect their fleas using either Sherman traps (H.B. Sherman Trap Inc., Tallahassee, Florida) or wire-mesh BTS traps (Besançon Technical Service, Besançon, France). To ensure public health and sanitary standards, all trapped rodents are humanely euthanized. Adult fleas are extracted by fur brushing and a homemade flea vacuum is used to collect any fleas that escape during the brushing process. Collected fleas are kept alive and transported to the laboratory in clear 2 l glass jars containing sterilized rice bran and larval food [16,32]. Bioassay is conducted on the subsequent generations of fleas following rearing in the insectary.

Adults of both sexes were randomly collected from rearing jars and divided into groups of ten and exposed to insecticide in 18 cm long glass test tubes containing a single strip of insecticide treated paper (1.5 cm × 6 cm). These papers impregnated with a mixture of inert organic carriers (oil and acetone) and insecticide were provided at a dose recommended and approved by the WHO. For the case of Madagascar, they were usually purchased at Vector Control Research Unit of University Sains, Malaysia, or prepared by the investigators. The tests were carried out in a room with controlled temperature (25 ± 2 °C) and relative humidity (80 ± 5%). Six batches of 10 adult fleas were used per test: four batches were exposed to insecticide impregnated papers while two batches were exposed to papers impregnated with a mixture of oil and acetone only, to serve as controls. The dead or paralyzed fleas were counted at defined time intervals during the diagnostic exposure time. After exposure, the impregnated papers were replaced by clean filter papers of the same size and fleas were kept at the same controlled conditions during 24 h, after which final flea mortality rates (both for control and test fleas) were recorded. The results were interpreted according to WHO criteria as previously mentioned [39].

#### Data analysis following susceptibility bioassay.

Different methods have been used to evaluate flea susceptibility to insecticides in Madagascar. Our analysis focused on bioassay data extracted from six scientific papers published between 1998 and 2022 with comparable methodologies [16,32,34,36,72,73]. These publications reported insecticide testing involving predetermined concentrations and diagnostic times, with susceptibility status interpreted from mortality rates following WHO thresholds [39]. All conducted flea susceptibility tests followed WHO standards for flea insecticide-coated paper tests. The database included a total of 262 independent insecticide susceptibility tests conducted on the primary plague vector, *X. cheopis*. A total of 11,666 *X. cheopis*, collected in 57 locations (i.e., 57 populations) were used for bioassays to establish phenotypical resistance status.

#### Active ingredients.

Thirteen active ingredients belonging to major insecticide families were tested (Table 2). Overall, synthetic pyrethroids were the most tested insecticides (55.7% of all test reports), represented by six active ingredients (deltamethrin, cyfluthrin, alphacypermethrin, etofenprox, permethrin and lambdacyhalothrin). Organophosphates were represented by three active ingredients namely fenitrothion, malathion and pyrimiphos-methyl, and represented 16.4% of all bioassays. Carbamates, represented by bendiocarb and propoxur, accounted for 14.5% of the bioassays. Bioassays involving Organochlorines, represented by DDT and dieldrin, represented 13.4% of the dataset. The number of tests conducted for each active ingredient are presented in Table 2, with deltamethrin being the most tested, followed by fenitrothion and permethrin.

Over time, the bioassay protocol has experienced changes, especially regarding diagnostic concentrations. For instance, the initial study defining the eight-hour exposure time for deltamethrin, which was generalized for all pyrethroids, was based on a concentration of 0.025% [30]. This concentration was maintained in studies published in 1998 and 2000 [34,73], while published work starting from 2014 adopted a higher concentration of 0.05% [16,32,36,72]. Several other changes in active ingredient concentrations are presented in Table 2. Nonetheless, with higher insecticide concentrations, we observed lower mortality rates, which may translate into the establishment of resistance for these active ingredients.

#### Spatial distribution.

**Fig 3:**
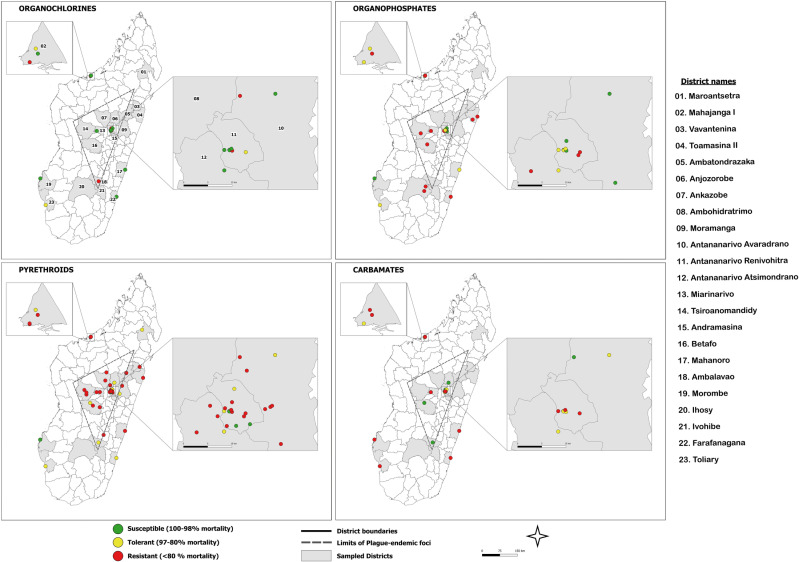
Geographical distribution of *Xenopsylla cheopis* susceptibility status for the four insecticide families commonly used to control flea vectors (studies published between 1998 and 2022). The map was generated with QGIS software (https://qgis.org/en/site/about/index.html). Administrative boundaries were downloaded from GADM: https://gadm.org/index.html.

Insecticide susceptibility data were recorded for 57 locations distributed in 23 districts of Madagascar (Fig 3). Among the 57 populations, 49 showed resistance to at least one insecticide from each of the four families. The geographical distribution of tested *X. cheopis* populations is shown in Fig 3. Most phenotypic tests have been carried out on flea populations collected from the Central Highlands of Madagascar and from the coastal town of Mahajanga, where human plague cases were reported in the 1990s [74]. Flea sampling for bioassay usually follows the occurrence of human plague cases but also occurs as part of surveillance programs resulting in some sampling sites in districts that never reported human plague cases. Interestingly, insecticide resistance to organophosphates and carbamates was recorded from flea populations from these regions (Fig 3). For instance, in Ihosy district, where plague cases have never been detected, insecticide resistance to fenitrothion was reported [16] (Fig 3). While most human plague cases were in rural areas, some surveillance programs included sampling in urban areas and detention centers outside of plague endemic foci where resistance to the majority of insecticides tested was also reported [72].

## Trends in resistance prevalence

**Fig 4:**
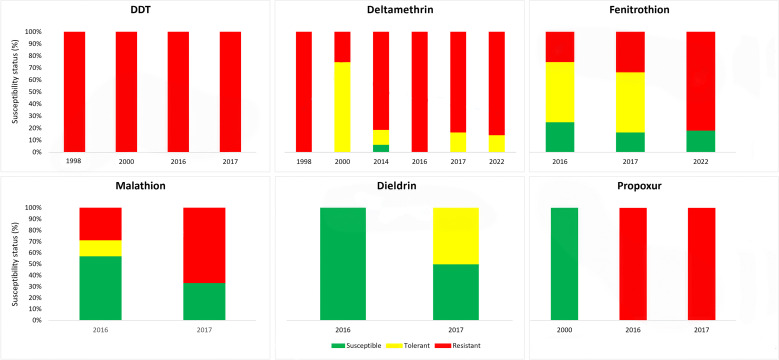
Evolution of flea resistance to the insecticides used for vector control in Madagascar. Susceptibility status expressed in % represents the proportion of bioassay that were concluded with susceptibility, tolerance or resistance. For instance, 100% of the bioassays conducted with DDT were concluded with resistance.

Results from susceptibility bioassays play an important role in shaping the plague vector control strategy in Madagascar. In this review, we analyzed bioassay results published from 1998 to 2022. After the last publication of results in 2000, there was a 14-year hiatus in publication of flea insecticide susceptibility data in Madagascar. Nonetheless, insecticide bioassays have been continuously conducted on fleas collected from plague foci, and the results have been recorded in institutional internal reports. Many of the insecticide tests were conducted several years before their publication in peer-reviewed journals [32,36,72]. For each publication examined, the active ingredient for each bioassay reflects the epidemiological context in which the flea samples were collected (Fig 4), usually flea susceptibility to the insecticide adopted by the NPCP at the time of the study (Fig 1), in addition to other active ingredients proposed as alternatives. For instance, the 1998 publication reports susceptibility of fleas to deltamethrin [34], after the failure of this product to give satisfactory results following an epidemic in the urban area of Mahajanga [74]. Even though resistance to deltamethrin has been demonstrated in fleas from a small number of urban areas in Madagascar, this insecticide has been used for plague control, and the report published in 2014 demonstrated widespread resistance to this compound in numerous populations collected in various time points and regions [32].

Deltamethrin was the most studied active ingredient from all articles published from 1998 to 2022, with only two bioassays showing susceptibility outcomes [32]. Resistance prevalence describes the proportion of bioassays with resistance as an outcome (Table 2). The lowest resistance was reported in 2000 (25%) from flea populations in some rural areas of the Central Highlands of Madagascar [73]. The resistance prevalence increased progressively throughout the years, with 100% of tested populations resistant to deltamethrin in 2022 [16]. In addition to deltamethrin, other pyrethroids were tested. Among the 146 tests involving pyrethroid insecticides, resistance was recorded in 114 bioassays (78.1%), while susceptibility was recorded in six. All populations tested for lambdacyhalothrin, alphacypermethrin and etofenprox were resistant, and 33.3% were resistant to cyfluthrin. For deltamethrin, resistance was 81.4%.

Several bioassays involving eleven other active ingredients, conducted on deltamethrin-resistant populations identified two organophosphates to replace deltamethrin [36]. In this review, malathion and fenitrothion had the lowest resistance among active ingredients used for plague control (Fig 4). The most recent publication on rat flea insecticide resistance status demonstrated that several populations were resistant to fenitrothion, along with deltamethrin and permethrin, five years after routine deployment of this organophosphate against plague vectors [16]. Overall, bioassays involving organophosphates showed that resistance prevalence was 52.0% for fenitrothion and 42.9% for malathion. None of the populations tested for pyrimiphos-methyl were resistant in bioassays conducted in 1998, but this insecticide has not been tested since.

Levels of organochlorine resistance have been heterogenous from 1998 to 2022 (Fig 4). Resistance to DDT was demonstrated before the 1990s [3,29]. The average mortality rate obtained with DDT never exceeded the 44.5% published in 2000 [73], and subsequent publications consistently demonstrated resistance for all populations tested (Table 2). The use of dieldrin has been discontinued since the 1980s [33], and articles published in 2016 and 2017 [36,72] reported very low resistance to this insecticide (Fig 4). In summary, although both DDT and dieldrin belong to the organochlorine family, 100.0% of bioassays involving DDT concluded with resistance, while no resistance to dieldrin has been demonstrated. However, the use of dieldrin is no longer recommended due to its high toxicity.

For carbamate insecticides, resistance prevalence was 70.0% for bendiocarb and 77.8% for propoxur. Propoxur was used for plague vector control from the mid-1970s until the end of the 1980s [33], and in 2000 [73], it was reported that all four populations tested were susceptible to this insecticide. Nonetheless, later publications reported a complete resistance to this carbamate [36,72].

## Resistance mechanisms

The knowledge of insecticide resistance mechanisms is essential for the development of insecticide-based strategies in vector control. Studies on resistance mechanisms of fleas focused mainly on the three genus *Ctenocephalides*, *Pulex* and *Xenopsylla* [75]. Flea resistance mechanisms have been extensively studied in the cat flea *C. felis.* Several molecular mechanisms, namely *Rdl*, *kdr*, *skdr* mutations [76–79], as well as biochemical assays aiming to detect an alterations in metabolic activity or increases in enzyme production, have been developed for this species [80]. Mutations occurring at two sites in the sodium channel (*kdr*) has been demonstrated in *P. irritans* [81,82] and *X. cheopis* [83]. Unfortunately, limited information was available on flea resistance mechanisms in Madagascar. The first genetic analysis on *X. cheopis* from Madagascar was performed to investigate two nonsynonymous single nucleotide polymorphisms (SNPs) in the voltage-gates sodium channel (VGSC) gene: L1014F and L1014H [84]. The L1014F mutation was present in all 25 populations; a more uncommon mutation, L1014H, was found in 12 collections. There was a significant positive relationship between the *kdr* allele frequency and the proportion of individuals surviving exposure to deltamethrin. This study provided insights into the genetic mechanisms contributing to pyrethroid resistance in *X. cheopis*, aiding in the evaluation of the effectiveness of this insecticide family for vector control in Madagascar. However, further research is needed to explore other resistance mechanisms to ensure comprehensive control strategies.

## Futures directions and recommendations

In Madagascar, plague vector control was conducted for decades mainly through insecticide use against adult fleas. Flea control was the first measure to be applied when a case of human plague was reported. The principal aim of vector control during plague outbreaks was to rapidly reduce flea density and subsequently, to stop disease transmission. However, plague control in Madagascar lacks preventive measures, with insecticides used only during reported outbreaks, in contrast to the ongoing control efforts against malaria mosquito vectors.

## Vector control improvement against indoor rodent fleas

Recent flea resistance to fenitrothion requires the search of an alternative insecticide to optimize flea control efforts during plague outbreaks in a context where resistance to deltamethrin is well established [16]. The present study revealed that many *X. cheopis* flea populations still expressed low resistance to malathion, when compared to other insecticides (Fig 3). Malathion has been among the insecticides recommended by WHO to control flea vectors during plague outbreaks [27]. Malathion was among the alternative insecticides proposed to replace deltamethrin, with bioassays resulting in mortality rates ranging from 57.5% to 100.0% [36]. Malathion (instead of fenitrothion) was the insecticide proposed by researchers using a compartmental model, which aimed to identify the most cost-effective intervention to prevent the expansion of plague epidemics in Madagascar [85]. However, since fenitrothion and malathion are both organophosphates, there is a risk for flea populations to develop multiple resistance.

For effective control, the evaluation of insecticide efficacy should consider not only the physical and chemical properties of the insecticide but also key parameters such as climatic and environmental factors, flea species distribution, abundance, and seasonality, which should be determined through field studies. Additionally, the assessment of new methods for flea vector control should be considered following WHO recommendations for plague vector management, including residual and non-residual insecticidal sprays [26].

### Vector control against outdoor rodent fleas

The endemic flea *S. fonquerniei* is associated with small mammals dwelling outdoors [17,18] and indoor insecticide treatment has little effect on fleas harbored by outdoor rodents [37]. The efficacy of bait stations, initially developed for rodent control in sylvatic plague contexts [42,43,86] has been investigated several times in Madagascar [37,44–46]. However, entomological data were inconsistent across studies, necessitating further investigation to guarantee safe and effective use of bait stations in the human plague context.

Trapping small mammals in a high-altitude primary forests of Madagascar demonstrated the presence of the plague bacillus in a natural wild habitat [19,87]. Some forest fleas commonly found belong to genera such as *Synopsyllus* and *Paractenopsyllus*, whose potential role in plague transmission remains uninvestigated. However, recent research showed the natural infection of *S. fonquerniei* into the cycle of sylvatic plague and rare presence of *X. cheopis* inside degraded forest, which could lead the connection between wild small mammals and human [19].

Systemic insecticide could be a promising alternative to target fleas on outdoor rodents [58]. The use of fipronil grain bait has shown significant promise in controlling flea populations and mitigating the sylvatic plague among black-tailed prairie dogs in the United State of America (USA) and *Rhombomys opimus* in the Central Asia [55,56,59,60]. Similar approach could be tested in Madagascar to target outdoor fleas. Additionally, a thorough understanding of *S. fonquerniei* ecology and distribution is necessary, along with careful consideration of human and domestic animal safety.

### Controlling the human flea

The human flea, *P. irritans*, is commonly found indoors in Madagascar [16,22,37]. In other countries, such as Tanzania, *P. irritans* is suspected to play a role in human-to-human *Y. pestis* transmission [88]. Despite having been found naturally infected with *Y. pestis*, *P. irritans* is not yet considered as plague vector and is not targeted by vector control efforts in Madagascar. In 2019, studies demonstrated the ineffectiveness of current vector control methods using fenitrothion powder to reduce the density of free-living fleas in houses, which are primarily *P. irritans* [37]. WHO recommended several insecticides, mainly as spray, to reduce indoor infestations of *P. irritans* [27], including malathion, which could be used synergistically in the fight against plague vectors. However, to implement an effective pest management program, resistance to insecticide in human fleas must be investigated. Due to nuisance and the medical importance of human fleas, target site insensitivity to pyrethroids in *P. irritans* has been investigated elsewhere [81,82]. Improving household conditions favoring *P. irritans* infestations has been suggested to minimize reliance on insecticides [89]. In addition, the vectorial capacity of the human flea to transmit the plague bacterium and its susceptibility status must be investigated before suggesting changes to the strategy to control plague transmission in Madagascar.

In addition to vector control recommendations based on specific flea ecology, future work should focus on updating the distribution of flea species. This includes monitoring changes in flea populations and their geographical spread, as well as assessing the impact of environmental changes on flea behavior and abundance. Understanding these dynamics is crucial for developing effective vector control strategies and anticipating potential outbreaks. Additionally, research should explore how climate change and habitat degradation influence interactions among fleas, hosts, and the pathogens they carry, to better predict and mitigate the risks of plague and other vector-borne diseases.

### Phenotypic resistance detection improvement

One problem that has arisen in testing insecticide resistance in flea populations in Madagascar has been the lack of susceptible flea strains to establish the diagnostic dose. Consequently, initial susceptibility tests were conducted using WHO-impregnated paper designed for mosquitoes [64,90]. A multicenter study of the doses and diagnostic times of insecticides recommended for vector control is needed for regular monitoring of resistance in flea vectors using susceptible strains [71]. Shortcomings of the filter paper-based bioassay center around limitations to take into account important abiotic and biotic factors, which could influence results of bioassays [91–93].

Abiotic factors such as temperature, relative humidity and lighting of the test rooms greatly influence the sensitivity of the fleas and the efficacy of the tested insecticides. The temperature and the relative humidity would impact not only the efficacy of the tested insecticides but also, the insect behaviors [91,92]. Biotic factors such as sex ratio and age are often hardly controlled during flea susceptibility testing, and can impact the bioassay results. Flea phenotypic testing exposes both sexes as both are hematophagous. According to Bossard et al., males cat fleas are more sensitive to insecticides than females [94]. Generally, the age of rat fleas has never been controlled during bioassays as opposed to mosquitoes. However, age seems to be a factor that determines resistance to chlorpyrifos in cat fleas: adults older than 48 h were more susceptible than newly emerged fleas [93].

The ability of exposed insects to avoid insecticide impregnated papers during bioassays has been described in some studies, not only on fleas but also on mosquitoes [95,96] highlighting the need for new tools for phenotypic resistance detection. For instance, an adaptation of the CDC Bottle assay but using Petri dishes, have proven effective for fleas [95,97]. While the currently used bioassay gave essential information regarding rat flea population susceptibility to various insecticides, the establishment of more accurate protocols, taking into account flea physiology and behavior toward toxicant exposure would greatly improve the accuracy of bioassays.

In the timespan covered in this review, we reported the bioassay results from 57 flea populations, distributed in 23 districts and 13 regions of Madagascar (Fig 3). Interestingly, our study revealed the insufficiency and absence of phenotypic resistance data from two active plague focus districts, Tsiroanomandidy and Ambositra [98]. The heterogeneous pattern of resistance may relate to the unique environment of each location, history of exposure to selective pressure, flea sampling circumstances, and gene flow. Overall, the study of phenotypic resistance in field populations was limited regarding population size and geographical distribution, but results were extrapolated to the entire geographic areas for selection of vector control measures. Resistance is genetically inherited and may induce fitness cost [99]. Environmental factors that may favor or limit a population expansion would likely impact resistance. Although research on flea population genetics remains limited, a study showed that Malagasy *X. cheopis* seems to be genetically and geographically structured [100]. Therefore, a better understanding of the population genetics of plague vectors in Madagascar is needed. Studies on gene flow, such as the spread of *kdr* resistance genes at different spatial scales (e.g., neighborhood/village, district, region), and their link to resistance mechanisms would provide valuable insights. Additionally, examining the impact of environmental factors would give new insight into the development of insecticide resistance.

Understanding the effects of various insecticides on a variety of arthropod species is challenging [101]. Research included in this review reported resistance in *X. cheopis* populations outside of plague epidemic areas, including areas where malaria control was being carried out. The detection of resistance in fleas collected outside of the plague areas suggests other insecticide pressures selecting resistant fleas in addition to the pesticides used in response to plague outbreaks. Attribution of flea resistance to insecticide used for malaria control is a compelling hypothesis that should be examined further [101].

Keeping farm animals inside homes is a common cultural practice in rural areas of Madagascar [89], and agricultural pesticides have been implicated in promoting resistance in some arthropods of public health importance [102–104]. Madagascar lacks data on the impact of household pest control, vector control against malaria, and use of insecticides in agriculture on the resistance of non-target insects to insecticides. Also, due to the ineffectiveness of insecticide treatments deployed during plague outbreaks against *P. irritans*, it is crucial to gather information on the prevalence of insecticide resistance in human flea populations. This data will help plan and initiate effective integrated pest and vector management interventions, taking into account their exposure to insecticide selection pressure.

### Resistance mechanisms investigation in fleas

Resistance mechanisms reflect the evolutionary pathway or selective pressures that have led to observed phenotypical resistance within targeted populations. Presently, changes in active ingredients for plague vector control are based solely on outcomes of mortality bioassay, underscoring the urgent need for a deeper understanding of the genetic and molecular mechanisms contributing to resistance. The recent detection of the knockdown resistance (*kdr*) mutation [84] and the existence of cross and multiple resistances among *X. cheopis* populations suggests that resistance mechanisms are at play. Investigating such mechanisms in Madagascar is essential to develop strategies to mitigate resistance in fleas involved in plague transmission.

## Conclusion

Through the compilation of published reports and scientific literature, we aim to highlight the current state of knowledge and challenges in vector control of plague in Madagascar. This review addresses concerns about the effectiveness of control methods, the susceptibility status and the development of insecticide resistance of rodent fleas. Ultimately, this compiled data can be used to develop research frameworks for plague vector control and identify areas for improvement in the management of bubonic plague in Madagascar and other countries.

## Abbreviations

**:**
DDT: dichlorodiphenyltrichloroethane
FI: flea index
HCH: hexachlorocyclohexane
kdr: knockdown resistance
SFI: specific flea index
SNPs: single nucleotide polymorphisms
VGSC: voltage-gates sodium channel
